# Preparation study of indocyanine green-rituximab: A new receptor-targeted tracer for sentinel lymph node in breast cancer

**DOI:** 10.18632/oncotarget.10204

**Published:** 2016-06-21

**Authors:** Bin-Bin Cong, Xiao Sun, Xian-Rang Song, Yan-Bing Liu, Tong Zhao, Xiao-Shan Cao, Peng-Fei Qiu, Chong-Lin Tian, Jin-Ming Yu, Yong-Sheng Wang

**Affiliations:** ^1^ School of Medicine and Life Sciences, University of Jinan and Shandong Academy of Medical Sciences, Jinan, 250200, China; ^2^ Breast Cancer Center, Shandong Cancer Hospital Affiliated to Shandong University, Jinan, 250117, China; ^3^ Basic Laboratory, Shandong Cancer Hospital Affiliated to Shandong University, Jinan, 250117, China; ^4^ Radiotherapy Department, Shandong Cancer Hospital Affiliated to Shandong University, Jinan, 250117, China

**Keywords:** sentinel lymph node biopsy, synthetic imaging agent, rituximab, indocyanine green, lymphoscintigraphy

## Abstract

An appropriate receptor-targeted tracer for sentinel lymph node biopsy (SLNB) was prepared. We combined the fluorescence tracer (Indocyanine green, ICG) with Rituximab (a chimeric human/murine monoclonal antibody targeting the CD20 antigen on the surface of lymphocyte) directly to produce a new tracer (ICG-Rituximab). When the new tracer drains to the lymph node, Rituximab will combine with CD20 receptor on the B-cell surface in the lymph node. If the statue of antibody-receptor connection does not reach saturation, the number of Rituximab is less than CD20. With this appropriate injection dose, the new tracer could only stay in sentinel lymph node (SLN) and make it imaging. Positive fluorescence SLN was detected 12 minutes after injection with no other organs imaging. The imaging of SLN was stable and clear for 20–24 hours. Due to SLN stained with more ICG than the lymphatic vessel, the fluorescence situation of SLN would be brighter than the vessel. The surgeon can detect the positive fluorescence SLN easily without following the fluorescence imaging lymphatic vessel. The results of our preliminary study showed that the new tracer might be useful for improving SLN imaging and worth further clinical study. SLNB with the new tracer could be a convenient method for detecting SLN and would become a standard performance in clinical practice.

## INTRODUCTION

Sentinel lymph node (SLN) is the first lymph nodes that receive lymphatic drainage from the primary tumor in breast cancer [1, 2]. Sentinel lymph node biopsy (SLNB) is now regarded as the standard of care in patients without clinical and radiological evidence of axillary lymph node metastases in early stage breast cancer [3–5]. Accurate detection of SLN metastases is an important step in staging, prognosis, and treatment for patients [6]. Currently, tracers for SLNB included dye, radiotracer, and fluorescence tracer (particle size 50–200 nm). Physical and chemical properties of these tracers including shape, coating material, and particle size influence the distribution of tracers in SLNB [7]. In clinical practice, the dye and the fluorescence tracer exists a problem that they could easily pass through the SLN and be uptake in distal or second echelon lymph nodes [7–9]. The dyed or fluorescence imaged lymphatic vessels must be found and followed to the first lymph node (defined as SLN) with these tracers [10]. This was the main reason for the low successful rate and high false negative rate with blue dye or fluorescence tracer alone for SLNB, even conducted by surgeons with sufficient clinical experience. Due to the short depth penetration (less than 1 cm) and the thin lymphatic vessel [11], it is hard to find the ICG fluorescence imaging lymphatic vessel in deeper tissue. Meanwhile, radiotracer has the drawback of radiation exposure and cooperation with nuclear medicine department, and its application has been limited by the nuclear medicine facilities in the developing country [7]. To prepare an appropriate receptor-targeted tracer for SLNB, the fluorescence tracer (Indocyanine green, ICG) was combined with Rituximab (a chimeric human/murine monoclonal antibody targeting the CD20 antigen on the surface of lymphocyte) directly to produce ICG-Rituximab. The biological property and safety limitation of the new tracer were evaluated.

## RESULTS

### Preparation of the new tracer

In the progress of preparation, the sediments were not identified in combination ratios of 1:3 and 1:4, but were identified obviously in combination ratios of 1:12, 1:16, and 1:32 in the reaction solution. And the dialysate was fluorescence positive detected by fluorescence imaging system in combination ratios of 1:3 but no fluorescence imaging in other ratios.

### Labeled rate of the new tracer

In the reaction system 1, the new tracer was found at the front of the test strip; in the reaction system 2, Rituximab and the new tracer were identified at base point. The ICG labeled rate of Rituximab was 100%.

### Molecular integrity and immune activity

**Figure 1:**
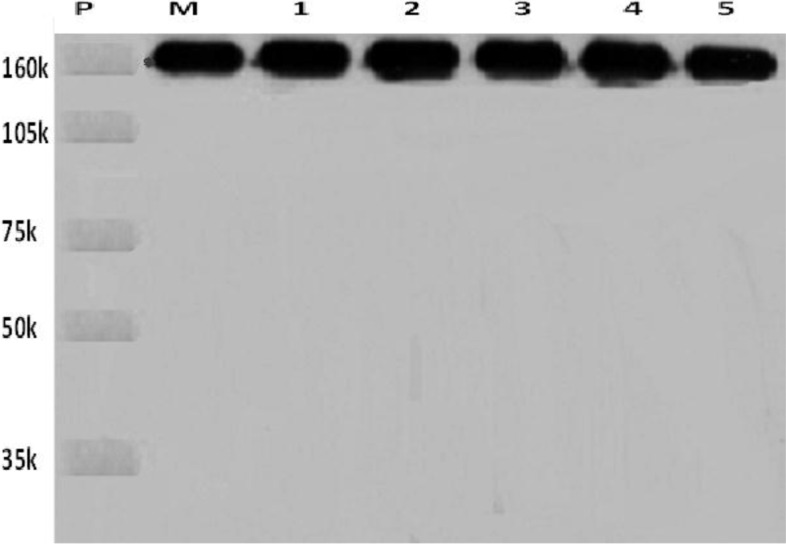
The new tracer was analyzed for molecular integrity by 8% sodium dodecyl sulfate-polyacrylamide gel electrophoresis P: Protein molecular weight marker; M: Rituximab protein; 1, 2, 3, 4, 5: New tracer ICG-Rituximab combination ratio in 3:1, 4:1, 12:1, 16:1, and 32:1.

**Figure 2:**
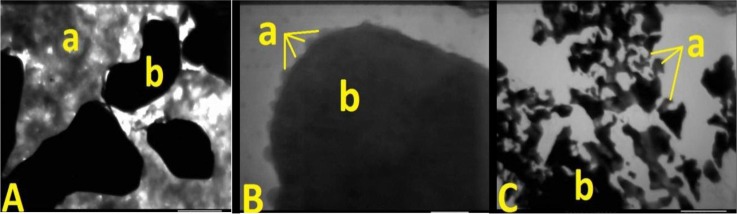
Scanning electron microscopy image of the new tracer, ICG (a) combined with the Rituximab (b) (**A**) is the combination ratios of 1:3, (**B**) is the combination ratios of 1:4, and (**C**) is the combination ratios of 1:12, 1:16, and 1:32. There are many uncombined ICG found in A and sediments observed in C.

The test of sodium dodecyl sulfate-polyacrylamide gel electrophoresis showed that the new tracers were positive results (Figure 1). The test of enzyme linked immune sorbent assay showed that the new tracer kept the immune activity of Rituximab. The scanning electron microscopy image of the new tracer was showed in Figure 2.

### Safety limitation test

The solution of the new tracer was identified no bacteria existence. The reaction solution of Limulus tests was clarified, so the new tracer was identified no pyogen. The acute toxicity test showed no mouse died and the local toxicity test found no skin reaction and no allergic reaction.

### SLN mapping and imaging

**Figure 3:**
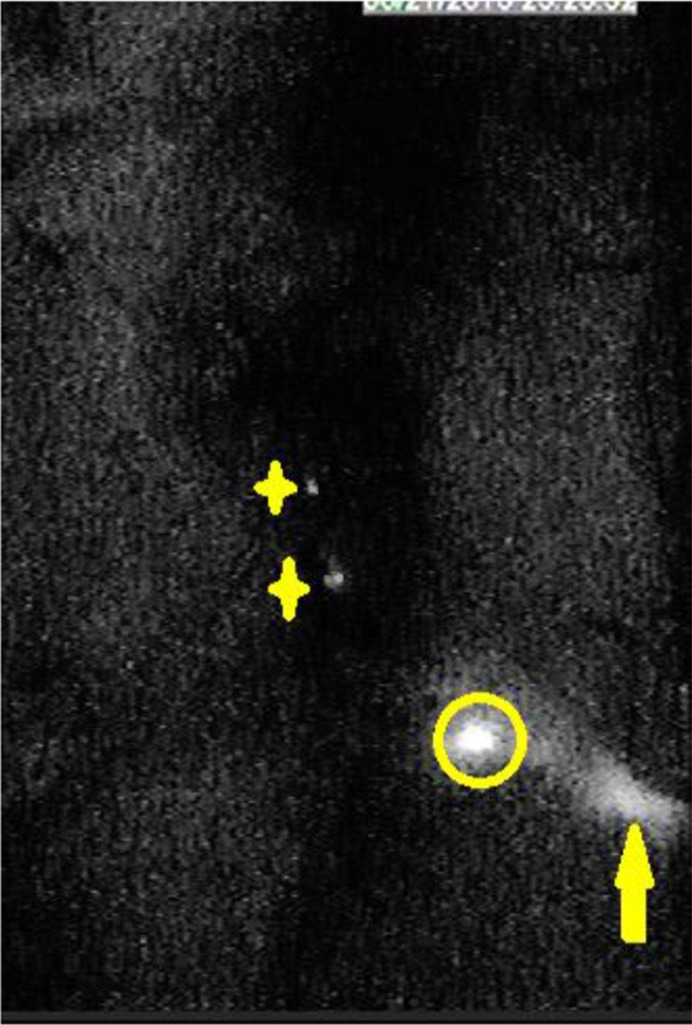
In the near-infrared fluorescence imaging, second echelon lymph nodes were found 20 minutes after injection with 50 μL and 100 μL (organs in abdominal and pelvic cavity were dissected; stars are second echelon lymph nodes in abdominal and pelvic cavity; circle is SLN; yellow arrow is injection point).

**Figure 4:**
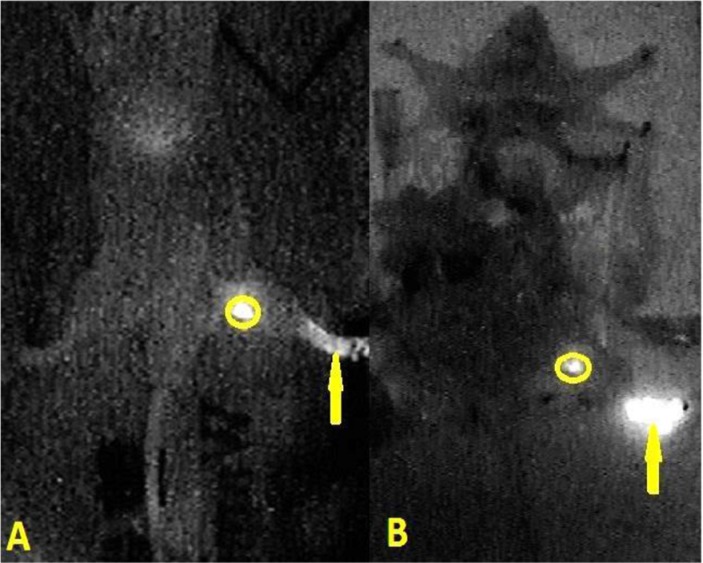
In the near-infrared fluorescence imaging, the situation of positive fluorescence SLN was identified 12 minutes (**A**) and 20 hours (**B**) after injection with 10 μL new tracer. (skin of leg and abdomen, organs in abdominal and pelvic cavity were dissected; circle is SLN; yellow arrow is injection point).

**Figure 5:**
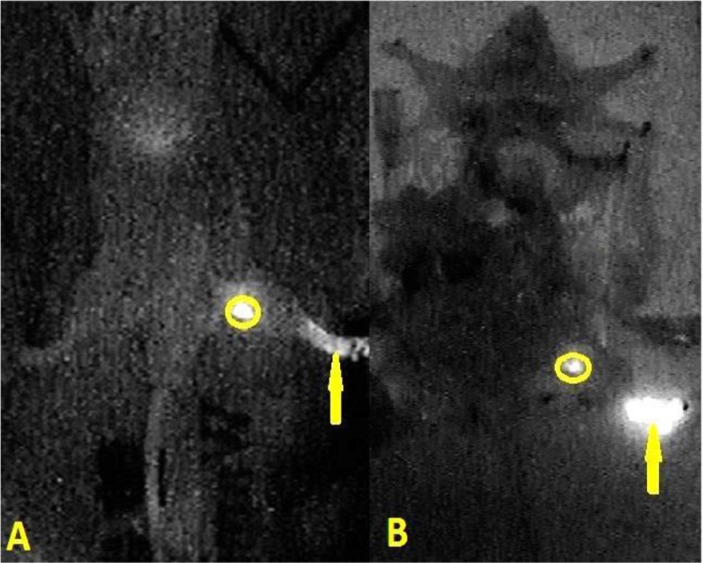
The fusion image of the near-infrared fluorescence and the cyclone storage phosphor system The location of SLN detected by the new tracer was consisted with the location of SLN detected by the radiotracer. (circle is SLN; yellow arrow is injection point).

In the group of 5 μL injection dose, the visualization rate of SLN was 60% (6/10) after injection. Second-tier lymph node imaging was found in 50 μL (30%, 3/10) and 100 μL (90%, 9/10) injection dose one hour after injection (Figure 3). In the group of 10 μL injection dose, only SLN imaging was identified after 12 minutes (*P* < 0.05), and no other nodes or organs imaging were found (10/10, Figure 4A). The imaging of SLN was stable and clear for 20–24 hours in this group (Figure 4B). The results of SLN mapping and imaging showed in Table 1. After radiotracer injection, SLN imaging was identified by gamma probe after 2 minutes and no other nodes imaging were found after 18 hours (10/10). The location of SLN identified by the new tracer (10/10) was accorded with by the standard radiotracer (Figure 5).

**Table 1:** The situation of SLN imaging after injecting with different dose

Dose Location	5 μL	10 μL	50 μL	100 μL
SLN	60%(6/10)	100%(10/10)	100%(10/10)	100%(10/10)
Second-tier LN	0%(0/10)	0%(10/10)	30%(3/10)	90%(9/10)

## DISCUSSION

Several studies have reported using near-infrared fluorescent ICG for SLNB in breast cancer, melanoma [12, 13]. The identification rate of SLN by ICG was better than by blue dye (*P* < 0.0001) [14–16]. But the removed number of SLN was higher for ICG than for blue dye and radioisotopes [16]. The study by Wishart and colleagues compared the ICG technique with the standard dual technique of blue dye and radioisotope, the results suggested that 17% of lymph nodes harvested by ICG were not SLN [7]. The reason for this is that ICG might travel to second echelon lymph nodes and it leads to unnecessarily excessive dissection of lymph nodes. Furthermore, the penetration depth of ICG fluorescence is less than 1 cm and the fluorescence situation of the lymphatic vessel is blurred [17, 18]. It is hard to find SLN by following the fluorescence imaging lymphatic vessel in deeper tissue. In our clinical practice, when the fluorescence imaging lymphatic vessel was cut off, the fluorescence ICG would leak out and pollute the surround tissue. It is very difficult to distinguish the fluorescence imaging lymphatic vessel and the polluted fluorescence tissue. Thus, it has to be conducted by the surgeon with sufficient clinical experience to find and follow the fluorescence imaging lymphatic vessel to find the first fluorescence lymph node (defined as SLN).

The radiotracer could be retained in the SLN for longer periods of time and achieve a high sensitivity detection rate of SLN. However, when SLN is close to the injection site, the radioactive emitted from SLN cannot be distinguished from the background signal. This is called for the shine-through effect of radiotracer [19, 20]. In addition, the high cost of the radioactive probe, the exposure of radiation, and the limitation of nuclear medicine facilities inhibit the application of this technique in clinical practice [21, 22].

In the study of hybrid radiotracer, ICG was combined with ^99m^Tc-nanocolloid to prepare ICG-^99m^Tc-nanocolloid, but it leads to the removal of a larger number of nodes. The reason for this is that ICG does not bind completely and permanently to the nanocolloid and the free ICG can drain into additional SLN [23, 24]. To find an appropriate receptor-target tracer for SLNB, several studies have been done to combine the traditional tracer (dye, ICG, and nanocolloid) with a special antibody (target CD20 or CD206 antigen in lymph nodes). Rituximab is an antibody targeting CD20 antigen on B-cell surface in lymph node. Malviya's study showed that Rituximab could be labeled with ^99m^Tc-nanocolloid without any modification in its biological activity and specificity for CD20 receptors [25]. The study of ^99m^Tc-carbonyl-DTPA-Rituximab also showed that Rituximab was successfully labeled with ^99m^Tc using the carbonyl core and tested in Wistar rat foot pad model for its suitability as a SLN detection tracer [26, 27]. However, the hybrid radiotracer still exists as the drawback of radiation exposed and its application is limited by the nuclear medicine facilities in the developing country.

In our study, the fluorescence tracer ICG was combined with Rituximab directly to produce a new receptor-targeted tracer (ICG-Rituximab) without radioactive burden for SLNB. ICG and Rituximab are safe to the human body, and both of them have been approved by FDA. ICG was approved to be used in intraoperative tumor imaging [28] and Rituximab was approved for the treatment of B-cell lymphoma in adults [29]. ICG is a small molecular weight of only 774 Da [30] and can bind rapidly to albumin [31]. Rituximab is composed with two heavy chains of 451 amino acids and two light chains of 213 amino acids with a molecular weight of 145 kD [32]. The histidine side chain of Rituximab has a binding affinity for active sulfur group of ICG [33]. In combination ratios of the new tracer with 1:12, 1:16, and 1:32, the sediments were identified obviously in the preparation and no SLN imaging was found after injection in animal model. In combination ratios of 1:3, the dialysate was fluorescence positive detected by fluorescence imaging system after purifying, free ICG exits in it, and second-tier lymph nodes imaging were found in animal model.

ICG has a character of low photo-stability and thermal stability [34, 35]. The studies about the stability showed that ICG would decompose and produce singlet oxygen when exposed to light [36, 37]. We used this character to identify ICG combined with Rituximab and on the surface of the antibody. When the narrow focused high energy electron beam in the vacuum environment of the scanning electron microscopy focused on the surface of the new tracer about 30 seconds, the ICG-Rituximab released some gases which were production of ICG. The scanning electron microscopy image of ICG-Rituximab showed that many uncombined ICG were found in combination ratios of 1:3 and sediments in 1:12, 1:16, and 1:32 (Figure 2). The sediment and the free ICG were not found in 1:4 (Figure 2), so the appropriate combination ratio of ICG and Rituximab might be 1:4. We performed further study to this combination ratio ICG-Rituximab. In the biological property study, Rituximab was combined with ICG efficiently and retained biochemical integrity, stability and biological activity in the combination ratio of 1:4 ICG-Rituximab. After identifying no bacteria existence and no pyogen of ICG-Rituximab, the toxicity test was done in mice. The acute toxicity test showed no mouse died and the local toxicity test found no skin reaction and no allergic reaction. While the standard dose of Rituximab recommended for the treatment of lymphoma is 375 mg/m^2^ for patient [38, 39], the dose of the new tracer used for SLNB is just 1/1000 of the therapeutic dose and the administration route has changed from intravenous injection to local injection. Thus, it is safe for the human body with local injection of ICG-Rituximab. Also, no toxicity had been found in the clinical studies of ICG (ICG-^99m^Tc-nanocolloid) and rituximab (^99m^Tc-carbonyl-DTPA-Rituximab and ^99m^Tc-nanocolloid-Rituximab) for SLN imaging [23–27].

**Figure 6:**
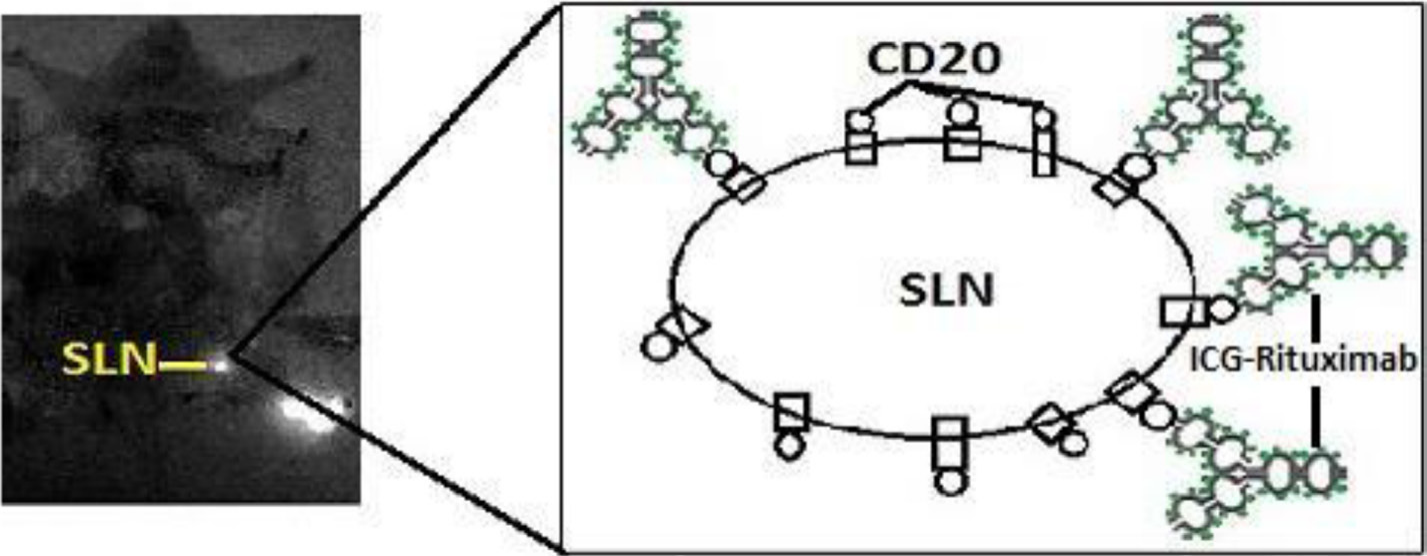
The statue of antibody-receptor connection does not reach saturation The number of Rituximab is less than CD20 on the surface of the lymph node. The new tracer could only stay in SLN and make it imaging in the near-infrared fluorescence imaging.

When the new tracer drains to the lymph node, Rituximab in the new tracer will combine with CD20 receptor on the B-cell surface in the lymph node. If the statue of antibody-receptor connection does not reach saturation, the number of Rituximab in the new tracer is less than CD20 on the surface of the lymph node, the new tracer could only stay in SLN and make it imaging. If the statue of connection reaches saturation, the number of Rituximab in the new tracer is more than CD20, the new tracer could drainage to second-tier lymph nodes continually (Figure 6). In the animal study of SLN imaging, only SLN imaging was found in the group of injection dose with 10 μL and second-tier lymph nodes were detected in other groups (50 μL and 100 μL) appeared 24 hours after injection. The appropriate injection dose of ICG-Rituximab for SLNB in mice is 10 μL (0.05 mg Rituximab, 0.0125 mg ICG), and its appropriate injection dose for human SLNB should be retested before its clinical application. Positive fluorescence SLN was detected 12 minutes after injection with the optimal injection dose. The imaging of SLN was stable and clear for 20–24 hours without other organs imaging. More ICG was gathered in SLN than lymphatic vessel, and the fluorescence situation of SLN would be brighter than the thin vessel. Surgeons just need to find and dissect the positive fluorescence SLN easily without following the weak fluorescence imaging lymphatic vessel. If the new tracer for SLNB is applied in clinical practice, the surgeon could prepare and evaluate the new tracer independently without coordination of other department and detect the positive fluorescence SLN easily without finding the thin fluorescence imaging lymphatic vessel. The clinical study of the new tracer was approved by the Institutional Ethics Committee of Shandong Cancer Hospital Affiliated to Shandong University and was undergoing in our breast cancer center. The results of our preliminary study showed that the new tracer might be useful for improving SLN imaging and worth further clinical study. SLNB with the new tracer could be a convenient method for detecting SLN and would become a standard performance in clinical practice.

In sum, the produce method of the new SLN tracer is simple, effective, and no radioactive burden. The new tracer could be used in SLN visualization for breast cancer patient. However, the appropriate injection dose for SLNB in the patient should be retested before its clinical application.

## MATERIALS AND METHODS

Rituximab was procured from F. Hoffmann-La Roche, Ltd (100 mg, Basel, Switzerland). Indocyanine green for injection was purchased from Dandong Yichuang Pharma, Ltd (25 mg, Dandong, Liaoning, PR China). Cyclone storage phosphor system was provided by the Department of Nuclear Medicine, Shandong University (Jinan, PR China). Dialysis tube was purchased from Spectrum Laboratories, Inc (Flat Width 24 mm, Molecular Weight Cut Off 3.5–5.0 kD, Rancho Dominguez, USA). Electrophoresis apparatus was procured from Bio-Rad Laboratories, Inc (Richmond, CA, USA). Silica gel plate for thin layer chromatography, enzyme linked immune sorbent assay (ELLAS) kit, and cell culture mediums were obtained from Sigma-Aldrich (Shanghai, PR China). All chemicals and reagents were of analytical grade.

### Animal model

**Figure 7:**
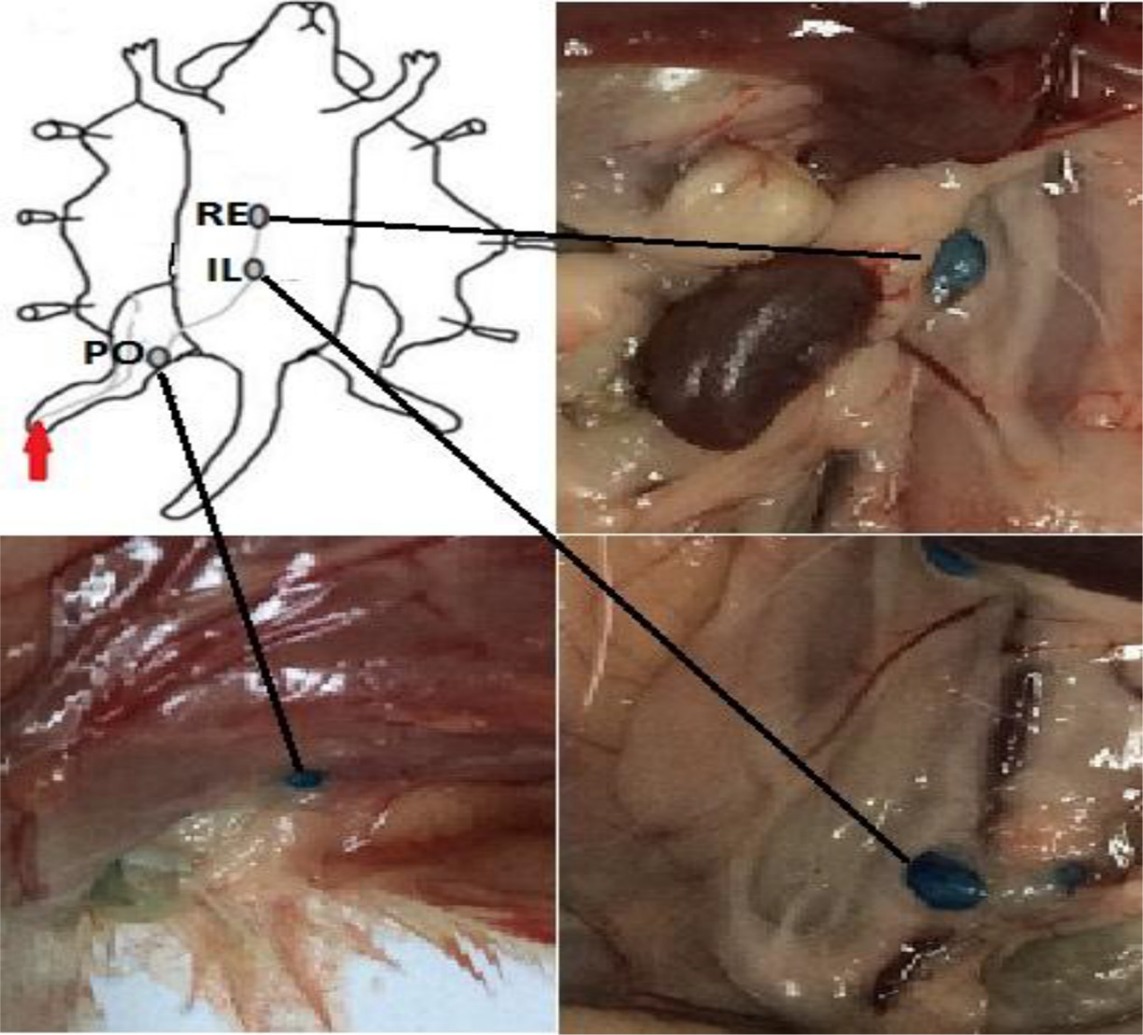
Lymphatic drainage of mouse footpad after methylene blue injection (red arrow) Lymph nodes staining with methylene blue were found from the ipsilateral popliteal lymph node (PO) to the iliac (IL) and renal (RE) lymph nodes after hind footpad injection.

Six-week-old male BALB/c mice were ordered from Shandong University Laboratory Animal Center (Jinan, Shandong, PR China). The BALB/c mice were bred under specific pathogen-free conditions in the Basic Laboratory, Shandong Cancer Hospital Affiliated to Shandong University for one week. After anesthesia with isoflurane, 50 μL solution of 1% (10 mg/mL) methylene blue was injected into the mouse hind foot paw [40]. This technique identifies lymph noeds involved in lymphatic drainage from the hind limb, with the ipsilateral popliteal, iliac, and renal lymph nodes consistently visualized within 15 minutes after injection (Figure 7). The popliteal lymph node was identified SLN and the other lymph nodes were identified the second-tier lymph nodes. All animal experiments were carried out after obtaining approval from the Shandong Cancer Hospital Affiliated to Shandong University Animal Ethics Committee.

### ICG near-infrared fluorescence imaging

The near-infrared fluorescence images were obtained using an MDM-I fluorescence imaging system (Mingde Biotech, Ltd, Langfang, PR China) consisting of photodynamic eye and TV monitor. The ICG absorbs light at a wavelength of 745 nm with emission of a fluorescent signal. The fluorescent signal was captured by the photodynamic eye which is composed of a series of light emitting diodes, a lens and a filter with the detector being a charge coupled device camera filtering out wavelength at 770–800 nm. The fluorescent signal appears on a TV monitor. The new tracer was transferred into a 0.2 mL eppendorf tube *in vitro* detection. The distance between the fluorescence detector and the tube was same at 1 cm before the tube. In the experiment of SLN imaging in mice, the distance between the fluorescence detector and the skin was approximately 10 cm at the middle before the body.

### Preparation of the new tracer

At ambient temperature and sterile condition (pH 7.5), 100 mg Rituximab was dissolved into 10 mL sterile injection water to get 10 mg/mL Rituximab solution, and 25 mg ICG was dissolved into 10 mL sterile injection water to get 2.5 mg/mL ICG solution. The solutions were then stored at 2–8°C shading condition prior to use. Rituximab solution was dissolved into ICG solution slowly. The reaction volume was 10 mL, and the solution was reacted for 5 minutes at ambient temperature. The concentration of ICG was 2.5 mg/L, and the mass ratios of ICG to Rituximab in the reaction solution was 1:3, 1:4, 1:12, 1:16, and 1:32. If sediment was found in the solution after combination reaction, the reaction solution should be centrifuged to remove the sediment (3,000 rpm, 20 minutes). After centrifugation, supernatant of the solution was purified using the dialysis tube. If no sediment was found, the reaction solution should be purified using the dialysis tube directly. The dialysis volume was 500 mL, and the solution was reacted at ambient temperature and shading condition. The volume ration of reaction mixture: sterile injection water was 1:200. The dialysate was changed every 2 hours, and detected by fluorescence imaging system to identify free ICG washed from reaction solution. After purifying, the synthesis of Rituximab combined ICG (ICG-Rituximab) was collected and stored at 2–8°C shading condition prior to use.

### Labeled rate of the new tracer

The labeled rate of new tracer was analyzed by instant thin-layer chromatography-silica gel. In the reaction system 1, the volume ratio of pyridine: ethanol: water was 5: 2: 1. The reaction system 2 was acetone. The critical of the reaction result: if using the reaction system 1, ICG would be at base point but the new tracer would be at the front of the test strip; if using the reaction system 2, Rituximab and the new tracer would be at base point but ICG would be at the front of the test strip.

### Molecular integrity and immune activity of the new tracer

The molecular integrity was analyzed by sodium dodecyl sulfate-polyacrylamide gel electrophoresis. Rituximab was regarded as a positive control to identify the molecular integrity of antibody in the new tracer. Scanning electron microscopy (provided by School of Physics and Electronic, Shandong Normal University) was used to evaluate the microstructure and morphology of the new tracer. The molecular immune activity was analyzed by enzyme linked immune sorbent assay. ELISA was performed in high protein binding capacity polystyrene plates.

### Bacteria detection

The new tracer was incubated in cell culture mediums at 37°C for one week to identify no bacteria existence.

### Pyrogen detection

The bacterial endotoxin of the new tracer was detected by Limulus tests. E. coli endotoxin was used as positive control, and sterile injection water was used as negative control. If the reaction solution was jelly like, the new tracer should contain pyrogen. If the reaction solution was clarified, the new tracer should no pyrogen.

### Toxicity test

Six-week-old male BALB/c mice were divided into five groups with four mice in each group. Five different mass ratios of ICG-Rituximab were injected intradermally into the mouse hind paw with a dose of 1.2 mg per kg and a dose of 12 mg per kg (10 and 100 times as human injection dose). After injection, the mouse was bred under specific pathogen-free conditions in the Basic Laboratory, Shandong Cancer Hospital Affiliated to Shandong University and observed for two weeks.

### SLN mapping and imaging

ICG-Rituximab was injected intradermally into the mouse hind paw with the dose of 10 μL, 50 μL, and 100 μL after anesthesia. Fluorescence images of the lymph node of mice were acquired by the MDM-I fluorescence imaging system. The images were monitored from 1 minute to 24 hours. The imaging time of SLN and other lymph nodes was recorded in detail. The standard SLN tracer (^99m^Tc-sulfur colloid, 10 μL, 10 μCi, 185kBq) was injected intradermally into the same hind paw of the ten mice which identified SLN by the new tracer (10 μL, 0.05 mg Rituximab, 0.0125 mg ICG). The location of inguinal SLN was identified by Neoprobe 2000 gamma detection system (Johnson & Johnson, New Brunswick, USA) two minutes after injection. Then, radiotracer images of inguinal SLN were acquired by the cyclone storage phosphor system. By this method, SLN identified by new tracer was observed whether consist with the radiotracer method.

### Statistical analysis

Analysis was performed with using the Statistical Program for Social Science (SPSS version 17.0) for Windows XP. Differences were considered to be statistically significant at *P* < 0.05 (*t*-test).
